# In dialogue with an avatar, language behavior is identical to dialogue with a human partner

**DOI:** 10.3758/s13428-015-0688-7

**Published:** 2015-12-16

**Authors:** Evelien Heyselaar, Peter Hagoort, Katrien Segaert

**Affiliations:** 1 Max Planck Institute for Psycholinguistics, Nijmegen, The Netherlands; 2 Donders Institute for Brain, Cognition and Behaviour, Nijmegen, The Netherlands; 3 School of Psychology, University of Birmingham, Birmingham, UK

**Keywords:** Language, Syntactic processing, Structural priming, Virtual reality, Human-computer interaction

## Abstract

The use of virtual reality (VR) as a methodological tool is becoming increasingly popular in behavioral research as its flexibility allows for a wide range of applications. This new method has not been as widely accepted in the field of psycholinguistics, however, possibly due to the assumption that language processing during human-computer interactions does not accurately reflect human-human interactions. Yet at the same time there is a growing need to study human-human language interactions in a tightly controlled context, which has not been possible using existing methods. VR, however, offers experimental control over parameters that cannot be (as finely) controlled in the real world. As such, in this study we aim to show that human-computer language interaction is comparable to human-human language interaction in virtual reality. In the current study we compare participants’ language behavior in a syntactic priming task with human versus computer partners: we used a human partner, a human-like avatar with human-like facial expressions and verbal behavior, and a computer-like avatar which had this humanness removed. As predicted, our study shows comparable priming effects between the human and human-like avatar suggesting that participants attributed human-like agency to the human-like avatar. Indeed, when interacting with the computer-like avatar, the priming effect was significantly decreased. This suggests that when interacting with a human-like avatar, sentence processing is comparable to interacting with a human partner. Our study therefore shows that VR is a valid platform for conducting language research and studying dialogue interactions in an ecologically valid manner.

## Introduction

The use of virtual reality (VR) as a method is becoming increasingly prevalent in behavioral studies in a wide range of fields, including navigation research (Tarr & Warren, 2002) and rehabilitation therapy (Rizzo & Kim, 2005). However, this new trend does not seem to be catching on as strongly in the field of psycholinguistics. This may be due to the assumption that humans do not interact with computers in the same way that they interact with other humans, making any behavioral measure of language interaction with a computer partner ecologically equivocal. However, in this study we aim to debunk this assumption by showing that language processing in interaction with a human-like virtual agent (“avatar”) is comparable to interactions with a human partner.

The assumption that humans do not interact with computers as if they have agency has already been shown to be false for interactions with desktop computers. Work by Nass and Moon (for review of their work see Nass & Moon, 2000) has repeatedly shown that humans attribute human-like characteristics to their desktop computer partner, the most unintuitive of these findings being the use of politeness when asked to evaluate the computer (Nass, Moon, & Carney, 1999). Participants would complete a task with Computer A and were afterwards asked to evaluate Computer A’s performance. If Computer A conducted this evaluation, the ratings were significantly more positive than if the evaluation was conducted by another computer (or on paper), suggesting that participants were polite to Computer A. These behaviors were also replicated for other human-like traits such as the attribution of social hierarchy (Nass, Fogg, & Moon, 1996) and even ethnic stereotyping (Nass, Isbister, & Lee, 2000). All of these were observed in participants who, during the debrief, agreed that “the computer is not a person and does not warrant human treatment or attribution.” The authors suggest that this might be due to a phenomenon referred to as *Ethopoeia*: humans are inherently social and therefore human-like rules also apply automatically and unconsciously in interactions with computers. This phenomenon therefore would predict that language behavior should also be no different when conversing with a computer.

VR is one step up from desktop computers as it offers an almost real-world-like immersive experience that a screen and keyboard cannot offer. The reason we are focusing on VR is that it offers an immersive 3D world that participants can move in and interact with, allowing experimental control over parameters that cannot be (as finely) controlled in the real world, and only limitedly so in desktop computers. What is particularly important for interaction research is that VR offers the ability to finely control interlocutor behavior in parameters that are nearly impossible to control in a confederate, an aspect that is particularly attractive for language research.

Experimental studies of language behavior use conversation-like tasks that allow for manipulations of isolated specific conversation characteristics. These conversation-like tasks usually use a constrained conversation in which participants interact with either a naïve participant or a confederate. These experiments allow researchers to focus on, for example, the turn-taking event (Finlayson & Corley, 2012; Stivers et al., 2009). the role of the dialogue partner (Branigan, Pickering, Pearson, McLean, & Nass, 2003). and characteristics of the social interaction (Balcetis & Dale, 2005). In the turn-taking literature, more and more emphasis is being put on the role of subtle cues, such as intonational or lexico syntactic cues, to signal when a partner can begin preparing their response. Looking into the roles of these cues is complicated with a confederate, as the cues need to be exactly the same with each participant or manipulated to ensure accurate millisecond precision. Additionally, studies have shown that the opinion you have of your partner can influence how you comprehend and produce language. These social cues can be as subtle as interacting with an in-group member compared to an out-group member (Unger, 2010), having similar political views to your partner (Weatherholtz et al., 2014) or even participants that like the confederate more (compared to other participants) exhibit significantly different language behavior (Balcetis & Dale, 2005). Replacing confederates with a recorded message without any physical presence is therefore unnatural and the participants may not respond naturally to these cues. However, it is possible that if we introduce a human-like computer, one with rich human-like facial features and expressions, the language behavior of the participant might be natural enough to be comparable to language behavior in human-human interactions, yet allow fine enough control over the characteristics of the computer to allow for experiments that cannot be conducted with a human confederate.

In this study, we put VR as a methodology to study language behavior to the test. We focused on syntactic processing (specifying the syntactic relations between words in the sentence), a core aspect of language production and comprehension, in the form of the commonly used syntactic priming task. Linguistic priming refers to the phenomenon in which an individual adopts the language behavior of their conversational partner (e.g., different word choices, different syntactic structures, etc.; also referred to as *alignment* or *accommodation*, Pickering & Garrod, 2004). Syntactic priming, specifically, refers to adapting your sentence structure, or syntax, to match that of your partner and has also been indicated to be influenced by the opinions you have of your partner (see above). Therefore, a syntactic priming task is an ideal candidate to test whether VR is a valid replacement for human partners in conversation studies.

Replacing a human partner with a virtual agent or robot is not novel (Blascovich et al., 2002). many researchers investigate how participants interact with machines (Bee, André, & Tober, 2009; Melo, Gratch, & Carnevale, 2014; Pena, Hancock, & Merola, 2009; Rehm & André, 2005; Rosenthal-von der Pütten, Krämer, Hoffmann, Sobieraj, & Eimler, 2013). However, these studies only compare behavior towards different types of avatars but make no connection to “natural” behavior (i.e., comparing to participant behavior when interacting with a human in the same situations). Recently, there have been a few studies comparing human and virtual agent behavior in the language domain (Branigan et al., 2003; Koulouri, Lauria, & Macredie, 2014; Pearson, Branigan, Pickering, & Nass, 2006) which have shown that participants prime *less* with a human-like computer compared to a human partner. These studies use *belief* to convince participants that their partner is human/not-human. Unfortunately, a follow-up study has shown that language behavior in a belief condition does not match that of face-to-face language behavior (Bergmann, Branigan, & Kopp, 2015). The study had participants interact with a desktop computer. In certain conditions the participants were told that they were interacting with another human seated in another room, in other conditions they were told they were interacting with a program. The results showed a different language behavior when the participants believed they were interacting with a human compared to a computer (similar to results shown by other human-computer language studies). However, if the participant was, in addition to the computer, presented with an animation of their apparent computer partner, their behavior did match that of human-human interaction. The authors explain this as: “when social cues and presence as created by a virtual human come into play, automatic social reactions appear to override the initial beliefs in shaping lexical alignment” (p. 9). Therefore, previous studies comparing human and computer interaction using only a belief manipulation may not be accurately measuring human-computer interactions. This emphasizes the importance of having an interlocutor present, which can be done using a desktop computer, but even more realistically when using VR.

In Experiment 1, we measured the magnitude of the priming effect when participants interacted with a human confederate and a human-like avatar with the hypothesis that priming behavior should be comparable. The human-like avatar had rich human-like facial expressions and verbal behavior. We conducted a follow-up experiment (Experiment 2) where participants interacted with the human-like as well as a computer-like avatar. The computer-like avatar had no facial expressions, her mouth movements did not match her speech, and all prosody was removed from her speech. As the magnitude of the priming effect is very susceptible to individual differences (Hartsuiker & Kolk, 1998). both experiments will be within-subject designs so that we can measure how priming behavior *changes* as a participant interacts with different partner types.

We expect that the priming effect when interacting with the computer-like avatar will be significantly less, since we hypothesize that any comparable effects observed between the human and human-like avatar partner seen in Experiment 1 are due to the humanness of our human-like avatar. To prevent any influence of belief on the results, we did not tell the participants that they would be interacting with a human-like and computer-like avatar. Instead we only informed them that the avatars were speech-recognition programs and that the participants are participating in a pilot study.

As the previous literature has suggested that the magnitude of the priming effect can be influenced by the opinion one has of their conversation partner, we also investigated whether we could replicate those results here and whether the degree of this influence is comparable between human and avatar partners. Previous human-computer priming studies have not included this potential co-variate. Instead most have looked at a correlation between perceived conversational success and priming magnitude (unsurprisingly; as conversational success is measured as understanding each other (Pickering & Garrod, 2004). there is a positive correlation; Koulouri et al. 2014). Therefore it will be interesting to see whether social influences on language behavior are also similar between human and avatar.

If we can provide evidence to support (1) that the magnitude of the priming effect is comparable between human-like avatars and humans, and that (2) these effects disappear when interacting with a computer-like avatar, we can confirm that VR is a valid method for replacing dialogue partners in language research. This could provide possibilities for new experiments in a wide range of subfields, such as turn-taking and social studies.

## Experiment 1

In this experiment we investigate whether participants prime to the same extent with a human as with an avatar partner, and whether the magnitude of this priming effect is modulated by social factors as self-reported by the participants. This is to confirm that language interactions with a virtual partner are ecologically valid and that VR experiments can be used to replace future experiments with human partners.

### Method

#### Participants

Fifty-three native Dutch speakers gave written informed consent prior to the experiment and were monetarily compensated for their participation. Five subjects were not convinced that the confederate was an ignorant participant and/or did not believe that the avatar was voice-recognition controlled (see Procedure) and were therefore a priori not considered part of the data set. Thus only 48 were included in the analysis (21 male/27 female, M_age_: 20.9; SD_age_: 2.5).

#### Statistical power

Statistical power was calculated using simulated priming data produced by the sim.glmm package (Johnson, Barry, Ferguson, & Müller, 2015) in R (R Core Development Team, 2011). For our simulated data set we assumed 25 repetitions per condition and 48 subjects. We assumed a 10 % increase in passive production following a passive prime compared to baseline condition. With a difference between avatar and human priming magnitude of 6 %, our simulated data set calculated a power of 0.751 with a 95 % confidence interval (CI) of 0.722–0.777 after 1,000 iterations.

#### Materials

##### Avatar

**Fig. 1:**
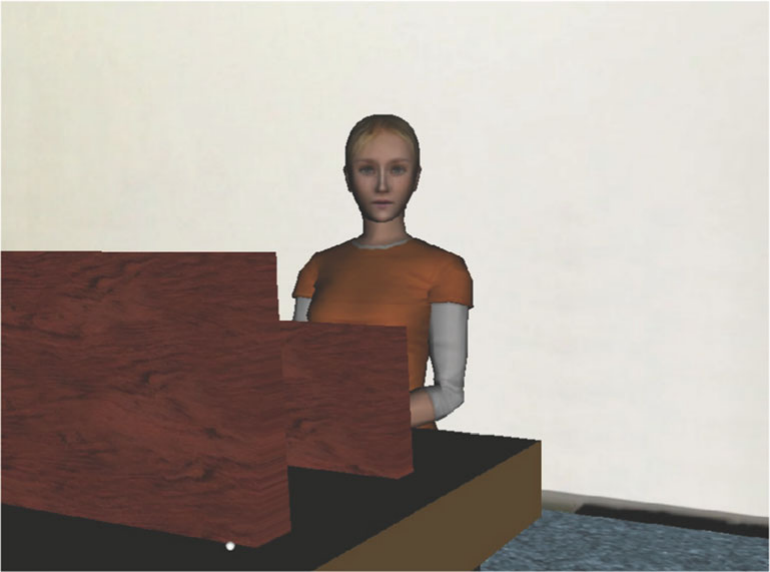
Avatar. The exterior of the avatar was identical for both avatar partners

The avatar was adapted from a stock avatar produced by WorldViz (“casual15_f_highpoly”) (Fig. 1). All the avatar’s speech was pre-recorded by a human female and played during appropriate sections of the experiment. The avatar’s appearance suggested that she was a Caucasian female in her mid-twenties, which matched the age and ethnicity of the Dutch speaker who recorded her speech. This Dutch speaker was not the same as the confederate.

##### Avatar characteristics

To choose the best and most human-like avatar, we collected data in a separate experiment (Heyselaar, Hagoort & Segaert, submitted). Six facial expressions (see Table 1) were judged by 30 participants not involved in the current study (13 male/17 female, M_age_: 22.5; SD_age_: 3.1) in categories such as humanness and familiarity, to see where they fell in the Uncanny Valley (Mori, 1970). The Uncanny Valley refers to the phenomenon in which human-like machines are perceived as less familiar than their less human-like counterparts. While we wanted to select the most human-like avatar, we needed to ensure that the avatar does not cross this threshold.

**Table 1:** Characteristics of the six pre-tested avatars

Avatar	Blink duration^1^	Smiling habit	Eyebrow habit
1	No blink	No smile	No movement
2	0.5 s	1/(3–5 s)	No movement
3	0.5 s	Constant smile	Constantly up
4	0.1 s (Normal)	No smile	1/(3–5 s)
5	0.1 s (Normal)	Dialogue-matched	1/(3–5 s)
6	0.1 s (Normal)	Dialogue-matched	Dialogue-matched

^1^Measured from the beginning of the closing movement to when the eye is fully open again

Avatar 6 was rated as significantly more human and less creepy than the other five (Tukey’s HSD, *p* < .05) and was therefore used in our study (henceforth “human-like avatar”). This avatar would blink once every 1–5 s (blink duration was 0.1 s), raise her eyebrows once every 1–5 s, and when not speaking she would smile once every 5–10 s. During speech, her eyebrow and smile behavior was explicitly programmed to match the content of her speech. For example, the avatar would raise her eyebrows when asking a question and smile when she was enthusiastic (“*Come, let’s play another round!*”). The influence of this blink and eyebrow behavior on agency is consistent with those of other studies looking into which characteristics support agency in the face (Looser & Wheatley, 2010; Tinwell, Grimshaw, & Williams, 2010). suggesting that these features (eyebrow movement and smile behavior) are necessary features to create human-like avatars. Additionally, her mouth movement was controlled by a program that matched her mouth’s openness to the pitch of the sound file. This created the illusion that her mouth movements were lip-synced to her speech.

##### Virtual environment

**Fig. 2:**
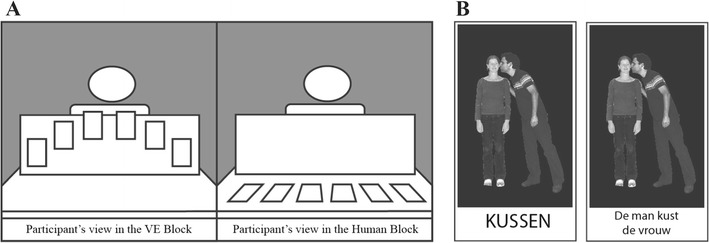
Set-up. (**A**) The experimental set-up from the view of the participant. The only difference is that in the virtual environment (VE) the cards were presented at the top of the divider, whereas in the Human block, the cards were laid out on the table. (**B**) The participant card (left) and confederate card (right). The participant card only showed the neutral verb associated with the photo, whereas the confederate card had a complete sentence written underneath. Here “to kiss” and “The man kisses the woman”

The virtual environment (VE) was a stock environment produced by WorldViz (“room.wrl”) adapted to include a table with a wooden divider (Fig. 2). This divider was comparable to the physical divider used in the Human block. To ensure that the amount of time spent looking at the partner’s face was the same between the Human and VE block, the divider was positioned so that while looking at the cards, the participant could not see the avatar’s face unless they explicitly lifted their head. This is the same in the Human block. The reason the cards were not placed on the table itself in the VE is due to the weight of the head-mounted display (HMD), which would cause an uncomfortable strain on the back of the participant’s head when the participant faces down. Having the participant’s face forward distributes this weight more comfortably.

The table in the VE matched in both dimension and position with a table in the physical world, such that participants could actually touch the “virtual” table.

The experiment was programmed and run using WorldViz’s Vizard software. Participants wore an NVIS nVisor SX60 HMD, which presented the VE at 1280 × 1024 resolution with a 60° monocular field of view. Mounted on the HMD was a set of eight reflective markers linked to a passive infrared DTrack 2 motion tracking system from ART Tracking, the data from which was used to update the participant’s viewpoint as she moved her head. It is known that this type of headset can cause dizziness and nausea due to the low frame-rate. However, for this study only two participants reported any discomfort. These participants were given a break and the option to continue; they both opted to complete the experiment. Additionally, a single reflective marker was taped onto the index finger of the participant’s dominant hand. This marker was rendered as a white ball in the VE, such that participants knew the position of their finger at all times. Sounds in the VE, including the voice of the avatar, were rendered with a 24-channel WorldViz Ambisonic Auralizer System.

##### Stimulus pictures

The pictures used in this task have been described elsewhere (Segaert, Menenti, Weber, & Hagoort, 2011). Our stimulus pictures depicted 40 transitive events such as *kissing, helping,* or *strangling* with the agent and patient of this action. Each event was depicted by a grey-scale photo containing either one pair of adults or one pair of children. There was one male and one female actor in each picture and each event was depicted with each of the two actors serving as the agent. The position of the agent (left or right) was randomized. These pictures were used to elicit transitive sentences; for each picture speakers can either produce an active transitive sentence (e.g. *the woman kisses the man*) or a passive transitive sentence (e.g. *the man is kissed by the woman*).

Filler pictures were used to elicit intransitive sentences. These fillers depicted events such as *running, singing,* or *bowing* using one actor. The actor could be any of the actors used in the transitive stimulus pictures.

Each card consisted of one stimulus picture with the relevant verb printed underneath. The cards were identical in the VE and Human block.

##### Questionnaire

The questionnaire used in this study is adapted (translated) from a questionnaire used in an earlier syntactic priming experiment by Weatherholtz et al. (2014). This study looked at the effect of political views on priming magnitude, and hence some questions were dropped as they were irrelevant for the current study (“My political views are usually conservative/liberal”) or if they did not have a direct Dutch translation (“The speaker appeared intelligent” and “The speaker appeared smart” both translate to the same sentence in Dutch). A previous study by Weatherholtz et al. (2014) also looked at how participants deal with conflict situations, and whether that could have an effect on how much they adapt their own behavior to match that of their partner. We included that question set as well. Of these, all of the original English questions were included.

All questions were phrased as statements, and the participants indicated the extent to which they agreed with each statement on a six-point scale (6 = I absolutely agree, 1 = I do not agree at all).

Participants were given questions relating to their opinion of their partner (hereafter *Relationship Questionnaire*, all questions listed in Table 2) after each condition. At the end of the experiment, participants filled in the questions asking how they dealt with conflict (hereafter *Conflict Questionnaire*, all questions listed in Table 3).

**Table 2:** Factor loadings for Relationship Questionnaire. Loadings greater than |0.4| are in bold as these items contribute most to the meaning of a factor. Loadings less than |0.1| are omitted for clarity

	Factor 1	Factor 2	Factor 3
	Likability	Selflessness	Shyness
I could be friends with my partner	0.72	0.37	0.19
My partner is similar to me	**0.73**	0.14	-0.13
My partner appeared generous	**0.53**	**0.62**	
My partner appeared intelligent	**0.84**	-0.12	**0.72**
My partner appeared selfish		**-0.92**	**0.87**
My partner appeared shy	0.15	0.21	**0.84**
My partner appeared enthusiastic	**0.53**	0.28	**0.72**
Proportion explained	0.46	0.30	0.24

**Table 3:** Factor loadings for the Conflict Questionnaire. Loadings greater than |0.4| are in bold as these items contribute most to the meaning of a factor. Loadings less than |0.1| are omitted for clarity

	Factor 4	Factor 5	Factor 5
	Ignore	Dominance	Compromised
I ignored the conflict and behaved as if nothing had happened	**0.93**		
I pretended there was no conflict	**0.92**		
I tried to find a middle ground	0.13	-0.11	**0.89**
I had a discussion with the other person to try to find a middle ground	-0.30	0.25	**0.73**
I insisted that it wasn’t my fault	0.13	**0.77**	
I kept pushing until the other person saw that I was right	-0.12	**0.83**	
I tried to convince the other person that my solution was the best	-0.23	**0.74**	0.20
Proportion explained	0.37	0.37	0.27

#### Task and design

All participants completed a language task probing syntactic processing in VE with an avatar (VE block) as well as in the physical world with a confederate (Human block; within-subjects design). The order of blocks was randomized and counterbalanced across participants. Partner type (human-like avatar vs. human) was used as an independent variable in the analysis.

Each block consisted of 228 trials (114 prime-target pairs). At the start of each block, the participant was presented with six cards, with the belief that the confederate/avatar had their own spread of six cards behind the divider (Fig. 2). The participant and the confederate/avatar would alternate in describing cards to each other. If the listener saw the card that was described by their partner as one of the cards on the divider, then both conversation partners would remove that card from the spread and replace it with a novel card from their deck (in VE this would happen automatically after the card was identified). This continued until all 228 cards were described. The confederate/avatar description would *always* serve as the prime for the participants’ subsequent target description.

The confederate’s deck was ordered identically to the participant’s deck, so the confederate/participant always had the card described to them. In the VE block, the avatar was programmed to randomly pick one of the participant’s cards to describe thereby assuring that the participant always had the card described to them. If the participant described the card correctly (see *Procedure* below) the avatar/confederate admitted to having the card the participant described. The same cards were used for both partner types.

The confederate’s deck of cards showed the stimulus picture but with a full sentence typed underneath, as such the confederate simply needed to read the sentence. Fifty percent of the transitive sentences described the picture in the passive tense, 50 % described it in the active tense. In VE, the avatar was programmed to use 50 % passives, 50 % actives.

The priming conditions were included in the analysis as independent variables. There were three priming conditions: baseline trials (intransitive prime followed by a transitive target), active priming trials (active prime followed by a transitive target), and passive priming trials (passive prime followed by a transitive target). However, as the participant was free to choose a card to describe, the chance existed that the participant would pick an intransitive card to describe in the target phase. These trials cannot be analysed in terms of active or passive syntactic structure. Therefore, to ensure an adequate number of trials in each condition, out of the 228 cards two-thirds were transitive and one-third were intransitive. *Post-hoc* analysis showed that there was an average of 24.7 (standard deviation (SD): 7.4), 28.3 (SD: 3.4), and 25.3 (SD: 3.2) trials in the baseline, passive, and active conditions, respectively, in the Human block and 20.7 (SD: 4.0), 24.6 (SD: 3.5), and 25.1 (SD: 4.0) trials in the baseline, passive, and active conditions respectively in the VE block. One subject was discarded as the difference in the proportion of passive prime exposure between the two blocks (Human: 0.40; VE: 0.65) fell 2.5 SDs outside the mean difference between blocks (mean: 0.03; SD: 0.09).

#### Procedure

Participants were informed that our goal was to compare how experiencing events differed in VE compared to the real world. To ensure that the participants felt that they were communicating with a program and not a programmer, they were told that it worked on voice-recognition, and hence no third party was necessary to operate the program. Questionnaires were handed out after each condition, as well as at the end of the experiment. Debrief questions were also handed out at the end of the experiment to see whether the participant believed the avatar to be independently operated and the confederate to be a naïve participant.

Responses during the syntactic priming task were manually coded by the experimenter as active or passive. An active sentence is one where the agent of the action is named first (e.g., *the woman kisses the man*) and such sentences were coded as 0; a passive sentence is one where the agent of the action is named last (e.g., *the man is kissed by the woman)* and were coded as 1. This way the data are ready to be entered into a logit analysis. An independent rater blind to the purpose of the experiment verified that the coding of a random sample of participants was done correctly (inter-rater reliability of 1). Target responses were included in the analysis only if (1) both actors and the verb were named correctly (as a sentence naming only one of the actors does not qualify as a transitive sentence) and (2) no unnecessary information was included in the description (which constrains the participants to using either an active or a passive description). We excluded 1.0 % (109 out of 10,929) of the target responses because they were incorrect.

#### Analysis

##### Questionnaire

As each participant filled in the Relationship Questionnaire twice (once for each partner type), we conducted a multivariate exploratory factor analysis on these results. For the Conflict Questionnaire, we conducted a principal components factor analysis. However, as this study only consists of a maximum of 199 data entries for the Relationship Questionnaire and only 95 for the Conflict Questionnaire, a value that is too low to conduct an accurate analysis, we combined our data with that of a similar study (Schoot, Hagoort, & Segaert, 2014) to boost the total data set to 310 for the Relationship Questionnaire (Kaiser-Meyer-Olkin Measure of Sampling Adequacy (KMO): 0.80; Bartlett's test of sphericity: χ^2^(21) = 611.48, *p* < .0001), and 155 for the Conflict Questionnaire (KMO: 0.60; Bartlett's test of sphericity: χ^2^(21) = 224.32, *p* < .0001). This was possible as the study by Schoot et al. (2014) also investigated how priming magnitude changed across one experimental session, and used the exact same questionnaire as the one used in this study. We used Jollife’s criterion (Jollife, 1972, 1986) as the cut-off criterion (eigenvalues < 0.7) and extracted three factors per questionnaire. Tables 2 and 3 shows the loading values for each of the extracted factors. Below each factor is the name we assigned to it, which we believe captures the theme of the factor best (i.e., the type of questions that contribute most to the meaning of the factor).

##### Mixed model analysis

The responses were analysed using a mixed-effects logit model, using the glmer function of the lme4 package (version 1.1.-4; Bates et al., 2012) in R (R Core Development Team, 2011). Target responses were coded as 0 for actives and 1 for passives. We used a maximal random-effects structure (Barr, Levy, Scheepers, & Tily, 2013; Jaeger, 2009). the repeated-measures nature of the data was modelled by including a per-participant and per-item random adjustment to the fixed intercept (“random intercept”). We attempted to include as many per-participant and per-item random adjustments to the fixed effects (“random slopes”) as was supported by the data. The full model included random slopes for *Prime* for the per-participant and the per-item random intercept. The correlations between intercept and slope for these random effects were between −1 and 1, suggesting that the model has not been over parameterized. Likelihood ratio tests were used to ensure that the inclusion of the remaining random slopes and random intercepts are justified.

Factorial predictors were dummy coded (all means compared to a reference group) and all numeric predictors were centered. We used Human as the reference group for *Partner Type,* and intransitives as the reference group for *Prime*.

### Results

#### Priming magnitude is the same between human and human-like avatar partner

**Fig. 3:**
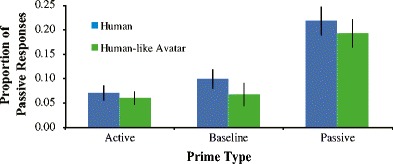
Proportion of passive responses per prime type for Experiment 1. As predicted, there are no significant differences in syntactic priming effects between the human and the avatar block. Passive production increased by 11.8 % for the human block and by 12.3 % for the avatar block following a passive prime compared to the baseline condition. In line with previous research, there were no priming effects for actives. Error bars represent standard error

Figure 3 summarizes the relative proportion of passive target responses after each prime structure. To test our first hypothesis that the priming magnitude should not be different between partner types, we ran a basic logit mixed model with only Prime * Partner Type as a fixed effect. The output is shown in Table 4.

**Table 4:** Summary for fixed effects in the mixed-logit model for passive versus active response choices between Human and Avatar

Predictor	Coefficient	*SE*	*Wald Z*	*p*	
Intercept (intransitive prime)	−3.11	0.29	−10.80	< .001	***
Active prime (AP)	−0.44	0.26	−1.72	.085	
Passive prime (PP)	1.27	0.20	6.26	< .001	***
Partner type (Human vs. Avatar)	−0.36	0.18	−1.96	.050	
AP * Partner Type	0.24	0.26	0.92	.359	
PP * Partner Type	0.22	0.21	1.01	.310	

Note: N = 6,931, log-likelihood = −2,004.6. * < .05  ** < .01  *** < .001

The negative estimate for the intercept indicates that in the baseline condition active responses were more frequent than passive responses. Following passive primes, more passive responses were produced compared to baseline (*p* < .001). Following active primes, there was no increase in active responses compared to baseline (*p* = .085). Neither active nor passive priming interacted with partner type (β = 0.24, *p* = .359; β = 0.22, *p* = .310 respectively), suggesting that the priming effect is the same in the Human and VE block. The main effect of *Partner Type* is almost significant (*p =* .050).

Looking at Fig. 3, this is most likely driven by the fact that there are marginally less passives produced when interacting with the avatar overall, regardless of prime type. Importantly, the priming effect is not significantly different between partner types (11.8 % for human partner, 12.3 % for avatar partner, *p* > .310).

#### Influences on the magnitude of the priming effect

To test our other hypotheses, we ran a mixed model in which we included all other measured variables, such as *Cumulative Passive Proportion, Order, Gender*, and all factors extracted from the questionnaire as well as interactions of all these factors with *Cumulative Passive Proportion and Prime*. *Cumulative Passive Proportion* is another way to present *Prime* but including a temporal element: the factor is calculated as the proportion of passives out of the total target responses produced on the target trials before the current target trial. A positive *Cumulative Passive Proportion* therefore suggests that the proportion of passives previously produced positively influences the probability of producing a passive on the current target trial. All categorical factors were treatment coded; the reference levels used were first block for *Order,* human partner for *Partner Type,* and intransitive (baseline) primes for *Prime.* We started with a full model (AIC: 3979.2, BIC: 4232.5), and performed a step-wise “best-path” reduction procedure (using the drop1 function in R) to locate the simplest model that did not differ significantly from the full model in terms of variance explained (AIC: 3967.8, BIC: 4152.6, *p* = .574). The collinearity was low (VIF < 1.90). This best model is illustrated in Table 5.

**Table 5:** Summary of fixed effects in the best model of influences on passive priming between Human and Avatar partners

Predictor	Coefficient	*SE*	*Wald Z*	*p*	
Intercept (intransitive prime)	−3.76	0.25	−14.82	< .001	***
Order 1 vs. 2	1.05	0.28	3.80	< .001	***
Cumulative passive proportion	2.74	0.45	6.02	< .001	***
Partner Type (Human vs. Avatar)	0.03	0.11	0.28	.501	
Likability	0.27	0.09	3.05	.003	**
Dominance in conflict	0.04	0.17	0.27	.800	
Dominance in conflict * Active prime	−0.28	0.13	−2.06	.042	*
Dominance in conflict * Passive prime	−0.21	0.13	−1.62	.105	
Cumulative passive proportion * Partner type	1.11	0.55	1.01	.012	*

Note: N = 6931, log-likelihood = −1,956.9. * < .05  ** < .01  *** < .001

**Table 6:** Summary for fixed effects in the mixed logit model for passive versus active response choices between Human-Like and Computer-Like Avatar

Predictor	Coefficient	*SE*	*Wald Z*	*p*	
Intercept (intransitive prime)	−3.48	0.35	−10.07	< .001	***
Active prime (AP)	−0.37	0.28	−1.33	0.18	
Passive prime (PP)	1.38	0.25	5.43	< .001	***
Partner type	−0.06	0.18	−0.33	0.74	
AP * Partner Type	−0.00	0.26	−0.02	0.99	
PP * Partner Type	−0.47	0.22	−2.13	.033	*

Note: N = 6627, log-likelihood = −1743.4. * < .05  ** < .01  *** < .001

The model shows significant contributions to passive production from *Order, Cumulative Passive Proportion, Likeability,* and *Dominance*. We will address each of these contributions in turn.

##### Order

The model shows a significant main effect of order. Specifically, there were significantly more passives produced in the second block (16.6 % of all responses) compared to the first block (7.5 % of all responses). This could be due to the fact that the participants have not interacted with an avatar before, and have also never completed the six-card priming task before and therefore might require some time to get their bearings. As the partner types were counter-balanced, this does not influence our main findings.

##### Cumulative Passive Proportion

**Fig. 4:**
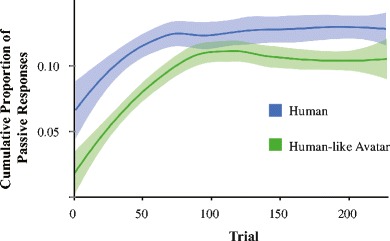
Cumulativity of passive responses for Experiment 1. The proportion of passive responses produced increases for both partner types over the course of the block. Mixed models show that there is a significant difference between the probability of producing a passive response between human and avatar blocks (*p* = .012). This is most likely due to the lower starting point of the avatar partner. The learning curve (between trial 0 and 75) is equally steep for other partner types

The current priming literature suggests that priming occurs due to implicit learning; the proportion of passives produced by the participant increases as a function of time. *Cumulative Passive Proportion* was calculated as the proportion of passives out of the total transitive responses produced before the current trial; Fig. 4 illustrates a similar increase over time for the human and avatar partner. Although the model shows that this effect is significantly different between partner types (*p* = .012), this is most likely driven by the fact that there are less passives produced overall with the avatar partner compared to the human partner.

We evaluated the influence of the *Cumulative Passive Proportion* on the syntactic response choice during the subsequent trial. Our mixed model analysis shows that a higher *Cumulative Passive Proportion* significantly increases the probability of a passive being produced. In other words, there is a cumulative effect of syntactic priming (i.e., the more passives produced, the stronger the effect) providing evidence for implicit learning as a mechanism for syntactic priming.

##### Likeability

**Fig. 5:**
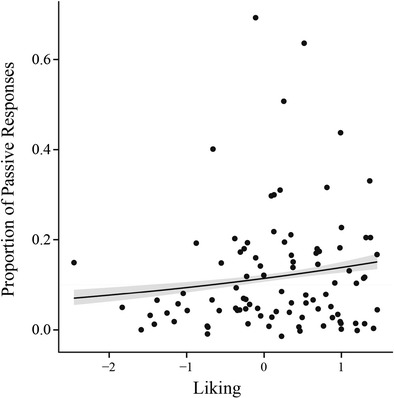
Main effect of liking on Passive Production. The more likeable the participant rated their interlocutor, the more passive responses the participant produced with that interlocutor (*p* = .003). This effect was not significantly different between human and avatar partner

The questionnaire we used is directly adapted from the questionnaire used in an earlier syntactic priming experiment by Weatherholtz et al. (2014) and would thus be expected to provide the closest means of comparison. However, Weatherholtz et al. (2014) found that *Likability* has a positive influence on priming magnitude, whereas we could find no such influence (Fig. 5): we only found a positive effect of *Likeability* on passive production, regardless of the prime type, such that the more the participant liked their partner, the more passives they produced. This effect was not significantly different for human versus avatar.

It is surprising that the effect of *Likeability* on priming magnitude is qualitatively different between the two studies, even though a comparable means of measuring liking was used. The effect of *Likeability* on priming magnitude should therefore be interpreted with caution. There are several differences in the set-up of the two studies, which suggests that social factors might mediate priming differently depending on the contextual and social environment, participant goals, etc. Further investigations will be necessary to elucidate how social factors influence priming magnitude.

##### Dominance in Conflict

**Fig. 6:**
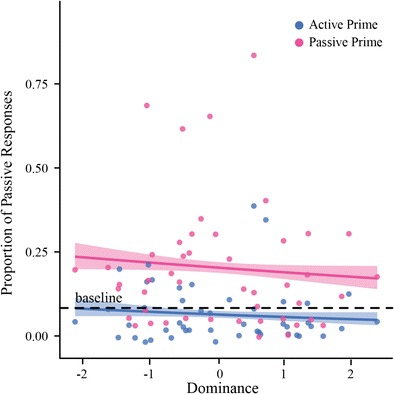
Effect of Dominance on Passive Priming. With increasing self-ratings of *Dominance in Conflict*, participants produced less passive responses compared to participants who rated themselves as less dominant in conflict situations. The model stated that there is a significant difference between how responses are effected based on their prime time (active vs. passive; *p* = .040); however, upon closer observation this effect may be influenced by the variability. Error clouds represent standard error

Our questionnaire also revealed a significant influence of dominance on passive production: participants who rated themselves as being more dominating when dealing with a conflict produced less passives. Just like *Order*, the lack of a significant interaction with *Partner Type* indicates that this was independent of whether the participant's partner was human or computer. The model suggests that *Dominance* has a significant influence on the passives produced following an active prime, but not a passive. This is depicted in Fig. 6. This figure shows a negative trend for passive production following both passive and active prime with increasing self-ratings of dominance in a conflict. Although the trend seems the same for both prime types, only active primes came out as significant in the model, most likely because the variability is lower (as indicated by the narrower error cloud).

### Intermediate conclusion

Our data show that our initial hypothesis was correct: priming magnitude is comparable between human and human-like avatar partners (12.3 % vs. 11.8 %). Investigating potential influences on priming magnitude revealed no significant effect of partner type, suggesting that these trends are identical between human and human-like avatar partner. In terms of social influences, we did not see a difference between partner types, and most only influenced the overall proportion of passives produced, but not on the priming effect itself.

We propose that this null-effect on priming magnitude is due to the humanness of our avatar, yet to be able to claim this unequivocally we need to show that the priming effect is not present when the avatar used is not human-like.

## Experiment 2

To determine whether the similarity in language behavior between the human and avatar condition is due to the perceived humanness of the avatar, we conducted a separate experiment in which language behavior was compared between a human-like and a computer-like avatar. In this experiment, the human-like avatar is the avatar used in Experiment 1. To create a computer-like avatar, we attempted to remove as much humanness as possible from the human-like avatar, i.e., it has no facial expressions, it doesn’t look at the participant, and all prosody was removed from the audio files.

### Method

#### Participants

Fifty-five native Dutch speakers gave written informed consent prior to the experiment and were monetarily compensated for their participation. None of these participants took part in Experiment 1.

Seven subjects did not believe that the avatar was voice-recognition controlled and were therefore *a priori* not considered part of the data set. Thus only 48 (22 male/26 female, M_age_: 22.08; SD_age_: 2.79) were included in the analysis.

#### Materials

##### Computer-like avatar

The exterior of the avatar matched that of previous experiments. However, all facial expressions were removed and when she spoke her mouth no longer matched the pitch of her speech; instead it opened and closed in a loop, very much like a fish. The avatar was also programmed to stare straight ahead, instead of always looking at the participant. To ensure that participants do not respond to the humanness in the audio files, the pitch range was set to 0 in all audio files, which caused all prosody to be removed. Regardless, participants on average gave the computer-like avatar a rating of 4.5 (out of 6; SD: 1.25) on how easy she was to understand, compared to a 5.6 (SD: 0.54) for the human-like avatar.

**Fig. 7:**
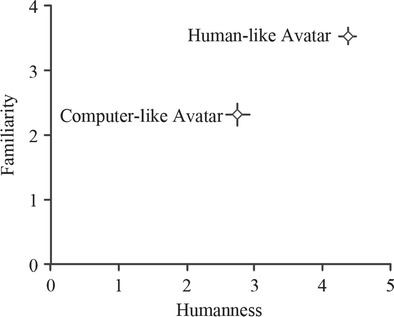
Humanness and familiarity ratings of the two avatar types. Ratings were given immediately after the encounter with the avatar, although participants were able to change their answer after they had been exposed to both. Error bars represent standard error. The computer-like avatar was rated as significantly less human (*p* < .0001)

To ensure that there is a difference between the human-like and computer-like avatars in terms of humanness, participants were asked to rate both avatars on their humanness and their familiarity. The results of this are shown in Fig. 7, illustrating that there is a significant difference between avatars in the humanness category (*p <* .0001, Paired *t*-test) but not in the familiarity category (*p* = .22, Paired *t*-test).

#### Task, design, and procedure

Task, design and procedure matched the VE block of Experiment 2. Participants were also asked to rate the avatars using the questionnaire from Experiment 1.We again manipulated priming (baseline, active, and passive prime) and partner type (computer-like vs. human-like avatar) as independent variables. We measured and analyzed syntactic priming choices for the target sentences.

*Post-hoc* analysis showed that there was an average of 20.4 (SD: 5.5), 24.7 (SD: 3.5), and 25.0 (SD: 3.0) trials in the baseline, passive, and active conditions, respectively, in the human-like avatar block and 20.1 (SD: 6.5), 23.5 (SD: 3.5), and 25.2 (SD: 3.4) trials in the baseline, passive, and active conditions, respectively, in the computer-like avatar block. No participants needed to be excluded in this experiment due to unbalanced passive exposure between blocks.

#### Analysis

We excluded 0.65 % (71 out of 10,861) of the target responses because they were incorrect (criteria described under *Procedure* of Experiment 1). For the logit mixed model, the same procedures were used as in Experiment 1, except for this model we included *Prime* and *Partner Type* as random slopes for the per-item random intercept. The per-subject random intercept is the same as Experiment 1 (*Prime* as a random slope).

### Results

#### Priming effect disappears with computer-like avatar

**Fig. 8:**
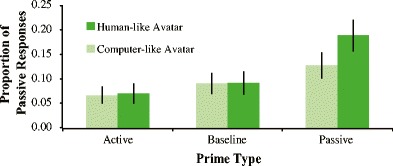
Proportion of passive responses per prime type for Experiment 2. There are significant differences in syntactic priming effects between the two avatar types (*p* = .033). Passive production increased by 9.5 % with the human-like avatar and only 3.7 % with the computer-like avatar following a passive prime compared to the baseline condition, which confirmed our prediction that participants primed less with the computer-like avatar as it is less human-like. In line with previous research, there were no priming effects for actives

Figure 8 summarizes the relative proportion of passive target responses after each prime structure. To test our first hypothesis that the priming magnitude should be different between partner types, we ran a basic logit mixed model with only Prime * Partner Type as a fixed effect. The output is shown in Table 6.

**Table 7:** Summary of fixed effects in the best model of influences on passive priming between Human-like and Computer-like Avatar partners

Predictor	coefficient	*SE*	*Wald Z*	*p*	
Intercept (intransitive prime)	−3.34	0.28	−12.05	< .001	***
Active prime (AP)	−0.29	0.22	−1.31	.192	
Passive prime (PP)	1.8	0.22	5.28	< .001	***
Cumulative Passive Proportion	3.37	0.57	5.91	< .001	****
Partner Type	−0.39	0.12	−3.28	.001	**
Dominance In Conflict	0.40	0.16	2.54	.011	*
Cumulative Passive Proportion * Partner Type	3.01	0.77	3.91	< .001	***

Note: N = 6607, log−likelihood = −1685.0. * < .05  ** < .01 *** < .001

The negative estimate for the intercept indicates that in the baseline condition active responses were more frequent than passive responses. Following passive primes, more passive responses were produced compared to baseline (*p* < .001). Following active primes, there was no increase in active responses compared to baseline (*p* = .18). As predicted, there was an interaction between passive priming and partner type (β = −0.47, *p* = .033), suggesting that participants primed less with the computer-like avatar compared to the human-like avatar.

#### Influences on the magnitude of the priming effect

To test our other hypotheses, we ran a mixed model in which we included all other measured variables, such as *Cumulative Passive Proportion, Order, Gender*, and all factors extracted from the questionnaire as well as interactions of all these factors with *Cumulative Passive Proportion and Prime*. All categorical factors were treatment coded; the reference levels used are the first block for *Order,* human partner for *Partner Type*, and intransitive (baseline) primes for *Prime.* We started with a full model (AIC: 3430.7, BIC: 3716.1), and performed a step-wise “best-path” reduction procedure (using the drop1 function in R) to locate the simplest model that did not differ significantly from the full model in terms of variance explained (AIC: 3416.0, BIC: 3572.3, *p* = .226). The collinearity was low (VIF < 1.54). This best model is illustrated in Table 7. The model shows significant contributions to passive production from *Cumulative Passive Proportion, Partner Type,* and *Dominance*. We will address each of these contributions in turn.

##### Cumulative Passive Proportion

**Fig. 9:**
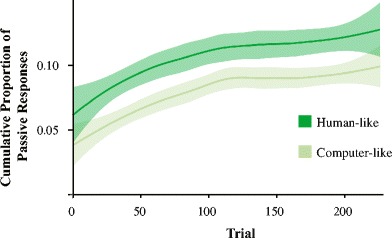
Cumulativity of passive responses for Experiment 2. The proportion of passive responses produced increases for both partner types over the course of the block. Mixed models show that there is a significant difference between the two avatar types (*p* = .0007)

Passive production over time is illustrated in Fig. 9, again showing that the proportion of passives produced increases over the course of the block for both partner types. *Cumulative Passive Proportion* is a significant predictor of syntactic response choice, similar to Experiment 1. Also similar to Experiment 1 is the significant difference between partner types (*p* <.001). However, looking at the shape of the curves, it appears that this interaction is driven by there being less passives produced overall in the computer-like avatar condition. The learning effect seems to be similar between partner types.

##### Partner Type

The main effect of *Partner Type* is driven by the fact that there are fewer passives produced when interacting with the computer-like avatar (9.7 % of all responses) compared to the human-like avatar (11.8 % of all responses).

##### Dominance in Conflict

**Fig. 10:**
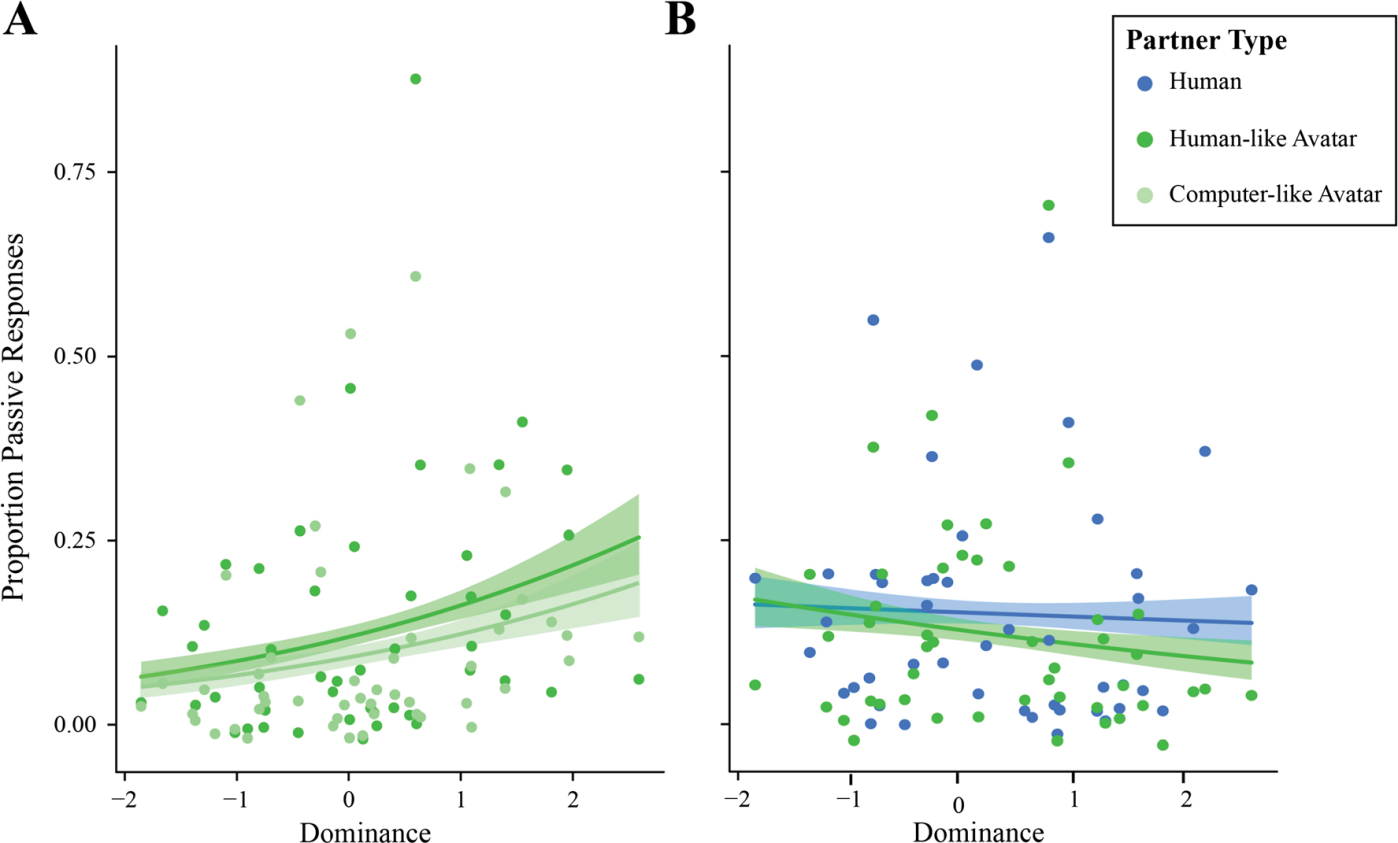
Effect of dominance on passive production per partner type. (**A**) The effects for Experiment 2. As self-ratings of dominant behavior in a conflict situation increase, the proportion of passive responses produced also increases. Curiously, **B** shows the opposite trend for Experiment 1. The human-like avatar is identical in both experiments, showing that this trend is most likely caused by the group make-up being different between experiments. This highlights the sensitivity of social factors to individual differences

Similar to Experiment 1, we find a main effect of self-rated *Dominance in Conflict* on passive production. This is illustrated in Fig. 10A. Contrary to Experiment 1, in this experiment participants who rated themselves as more dominant in a conflict situation showed increased passive production compared to participants who rated themselves as less dominant. Although the model did not show a significant difference between partner types, to ensure that this flip in results is not due to the computer-like avatar, we plotted the results per partner type. This shows that this effect is equally strong for both human-like and computer-like avatar. Plotting the same for Experiment 1 (Fig. 10B) again shows that it is not driven by one partner type; the results are exact opposites despite the conditions being identical (and the human-like avatar is identical) for the two experiments. This highlights the individual differences in social factors and their influence on syntactic choice.

## General discussion

**Fig. 11:**
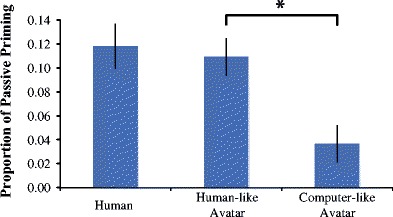
Priming magnitude per partner type. As priming with the human-like avatar was not significantly different between experiments (*p* = .85), the data are collapsed across experiments. Participants primed comparably with human and human-like avatar partners, but significantly less with the computer-like avatar (*p* = .03). As the only difference between the avatars was the humanness rating, the results suggest that the high priming magnitude of the human-like avatar is due to its perceived humanness

To validate whether VR is an ecologically valid method to study language in an interactive dialogue context, we measured syntactic processing during interactions with a human and two different avatar partners. To measure syntactic processing we performed the commonly used syntactic priming task and compared priming magnitude between the three conversation partners. Fully in line with our predictions, the results show comparable syntactic priming behavior when participants interacted with a human partner compared to an avatar partner with rich human-like facial expressions and verbal behavior (“human-like avatar”). When participants interacted with an avatar partner with all this richness removed (no facial expressions and no prosody in speech; “computer-like avatar”), this comparable syntactic priming effect disappeared. Our results therefore suggest that participants who are interacting with a human-like avatar elicit the same language behavior as if they were interacting with a human partner. We are attributing this finding to the humanness of the avatar, as when the experiment was repeated with an avatar that was rated as significantly less human compared to the human-like avatar, these effects disappeared (Fig. 11).

Three findings provide converging evidence that language behavior was similar when interacting with the human-like avatar compared to the human partner: (1) Syntactic priming effects were found when interacting with the human-like avatar as well as when interacting with the human partner and the size of these effects did not differ. In line with the literature, syntactic priming effects showed an inverse preference effect (syntactic priming effects for passives, not for actives (Bock, 1986; Ferreira, 2003) and these again did not differ between the two partner types; (ii) the influence of social factors on priming magnitude was not different between the human and human-like avatar partner; and (iii) in line with the literature, the chance of producing a passive increased as a function of time, suggesting the presence of implicit learning in our task (Jaeger & Snider, 2008).

In this study we show that priming magnitude significantly deteriorates when interacting with a computer-like partner. However, we are not suggesting that a partner is necessary for the priming effect to take place. Indeed, previous studies have shown that priming occurs without the physical presence of a partner (Levelt & Kelter, 1982; Stoyanchev & Stent, 2009) and therefore a conclusion one could draw is that the lack of humanness of the computer-like avatar is acting as an interfering factor that prevents the participant from priming. We suggest that if a study is in need of a physical presence, a human-like avatar can replace a human partner and will elicit human-like behavior whereas a non-human-like avatar will most likely inhibit naturalistic behavior. These things should be taken into consideration when designing the avatar partner.

In the current study, the human-like and computer-like avatar differ in that we used a computer-like or human-like voice, in addition to the use of facial expressions such as smiling and blinking habits. In this study we wanted to test two avatars that were as far removed as possible while still having them look identical. Therefore, based on these findings alone, we cannot conclude whether our findings are driven only by a difference in facial expressions, or also due to the use of different voices. However, in a follow-up study in which we only manipulated the facial expressions (the voice was identical for all avatars), we again find that perceived humanness determined syntactic priming effects: there was less priming for the avatar with no/less human-like facial expressions compared to the avatars with facial expressions (Heyselaar, Hagoort & Segaert, submitted).

In addition to looking at the differences in priming magnitude between the three partner types, we also investigated whether the same factors influence this increase in passive production behavior. Although we show an influence of social perception and personality of the participant on influencing passive production, our results were not consistent between experiments. Although the set-up and methodology was exactly the same between experiments (including the human-like avatar), we found differences in which factors influenced and how they influenced passive production, namely the factors *Likeability* (how likeable the participant found their partner) and *Dominance in Conflict* (how dominant participants rate themselves when in a conflict situation). Although the influences of the factors differ between experiments, there was no significant influence of *Partner Type* on the magnitude or direction of the factors suggesting that this difference is purely due to the different participant groups used. This highlights the danger of using between-subjects designs to look at social influences, as the preferential make-up of one group does not always match the make-up of the other, as we highlight here. The big difference in the influence of social factors between experiments also highlights how susceptible these factors are to individual differences, even in groups of 48 participants.

Although this study only provides evidence for syntactic processing, it suggests the possibility that other language behaviors may also be consistent between VE and the real world. Syntactic processing is a core aspect of language, and occurs at a high level of sentence processing (Hagoort, 2005). suggesting that events that occur at earlier levels in language processing could also be tested using avatar partners. Indeed, evidence for speech rate and pitch adaptation with avatar partners has already been shown (Casasanto, Jasmin, & Casasanto, 2010). This opens pathways for the use of VR to investigate behavior in the field of psycholinguistics. With the commercialization of virtual reality machines, marketed for the average family (e.g., Oculus Rift) or anyone with a smart phone (e.g., the Cardboard app. by Google), we believe that the current financial limitation of building a virtual reality laboratory will not be an issue in holding back future research. Additionally, studies have shown that as long as there is a virtual presence, even if that is an animated avatar presented on a desktop, the behavior elicited by participants is comparable to their behavior when interacting with another human, compared to a desktop without an animated being (Bergmann et al., 2015). VR offers a lot more possibilities for animation, but for some studies the animation possibilities of desktop computers would already be sufficient.

Additionally, our results also span into the field of robotics. Robotics has largely been concerned with creating human-like, realistic robots without investigating if humans interact with them the same as they would towards another fellow human. Recent studies have already started to investigate which features of the robot are necessary to get users to attribute agency to them, and the results are consistent with what we have found in our current study: simple features are the key. For example, one study has shown that a robot will be rated as having agency because it cheats when playing simple games such as rock-paper-scissors or battleships (Short, Hart, Vu, & Scassellati, 2010; Ullman, Leite, Phillips, Kim-Cohen, & Scassellati, 2014). Our study can add to this new area that simple facial expressions such as random smile and eyebrow movement are enough to elicit human-like behavior towards human-like robots. Future studies can use VR as an easily executable yet systematic method to determine which features are necessary to elicit agency.

In summary, VR provides an important platform on which previously unanswerable questions can now be investigated, providing a controlled method that produces results comparable to those seen in human literature.
